# A Case of De Garengeot Hernia With Associated Appendiceal Inflammation

**DOI:** 10.7759/cureus.89638

**Published:** 2025-08-08

**Authors:** Holly E West, Faisal Nadeem, Sian Abbott

**Affiliations:** 1 General Surgery, Walsall Manor Hospital, Walsall, GBR

**Keywords:** appendix, de garengeot, femoral hernia, laparoscopic appendectomy, low‐approach herniorrhaphy, mesh repair

## Abstract

A De Garengeot hernia describes the rare occurrence of an appendix located within a femoral hernia sac. An incidence of appendiceal inflammation associated with a De Garengeot hernia is an even rarer surgical finding. A woman in her 70s presented to a district general hospital with a two-week history of a mildly tender right-sided groin lump. This lump was diagnostically confirmed by a computed tomography (CT) scan to be a De Garengeot hernia with associated appendiceal inflammation. The patient was managed surgically with a combined laparoscopic appendectomy, followed by an open low‐approach herniorrhaphy. Primary suture repair of the femoral ring was performed due to concerns about contamination and mesh-related infection. This case highlights the diagnostic challenges associated with groin hernias containing atypical contents. Additionally, it stresses the importance of considering secondary appendiceal inflammation due to mechanical factors and compression, instead of infective processes, in cases of groin hernias with atypical symptomology.

## Introduction

An appendix located within an inguinal or femoral hernia sac is an uncommon surgical finding. When found in an inguinal hernia, it is called an Amyand’s hernia, accounting for 1% of all inguinal hernias [1]. Acute appendicitis within such hernias is more uncommon, occurring in only 0.1% of cases [1]. In comparison, an appendix within a femoral hernia is even rarer, with reported incidences ranging from 0.5% to 5% of femoral hernias [2]. This finding is referred to as a De Garengeot hernia, first described by French surgeon René Jacques Croissant de Garengeot in 1731 [3]. Here, we present a case of a De Garengeot hernia with associated appendiceal inflammation, which is reported to have an incidence as low as 0.08%-0.13% [4] and was first described by Hevin in 1785 [5].

## Case presentation

A woman in her 70s presented to the surgical assessment unit at a district general hospital in Birmingham with a two-week history of a mildly tender right-sided groin lump that she had not previously noticed. She had initially consulted her general practitioner (GP), who arranged an ultrasound scan one week prior to her presentation in hospital. This scan reported a possible right inguinal hernia containing the appendix; however, no images were saved to allow further review and interpretation. As a result, she was referred to the surgical assessment unit by her GP.

On assessment in secondary care, the patient reported that the lump was causing mild discomfort and not interrupting her activities of daily living. She denied any further abdominal pain, bowel changes, vomiting, or systemic upset. Her past surgical history was significant for a total abdominal hysterectomy and unilateral salpingo-oophorectomy for benign pathology performed 40 years previously. She was otherwise well, with a past medical history of asthma, hypertension, and diverticular disease. The patient had a performance status of 1 and lived independently.

On clinical examination, an irreducible, tender mass was palpable below the right inguinal ligament. There was no cough impulse, and examination of the inguinal hernia orifice was unremarkable. The patient had a BMI of 24 kg/m^2^. The clinical impression from examination was of a femoral hernia. The patient was haemodynamically stable and apyrexial, with a white cell count of 9.0 x 10^9^/L and CRP of 1 mg/L.

**Figure 1:**
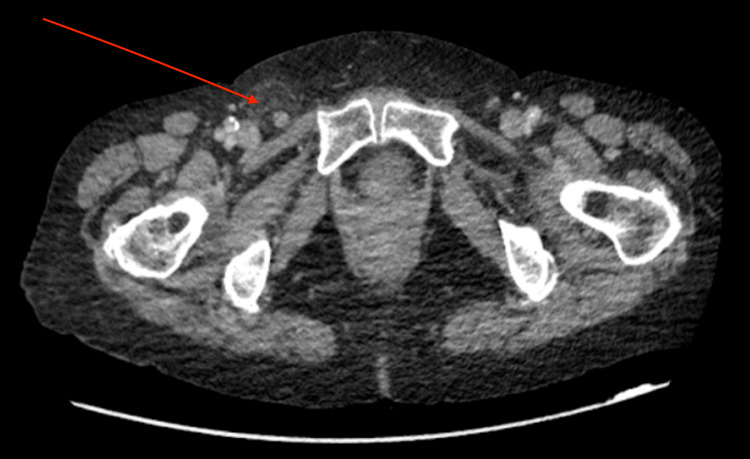
An axial section of a CT scan showing an inflamed appendix within a right-sided femoral hernia, indicated by an arrow

**Figure 2:**
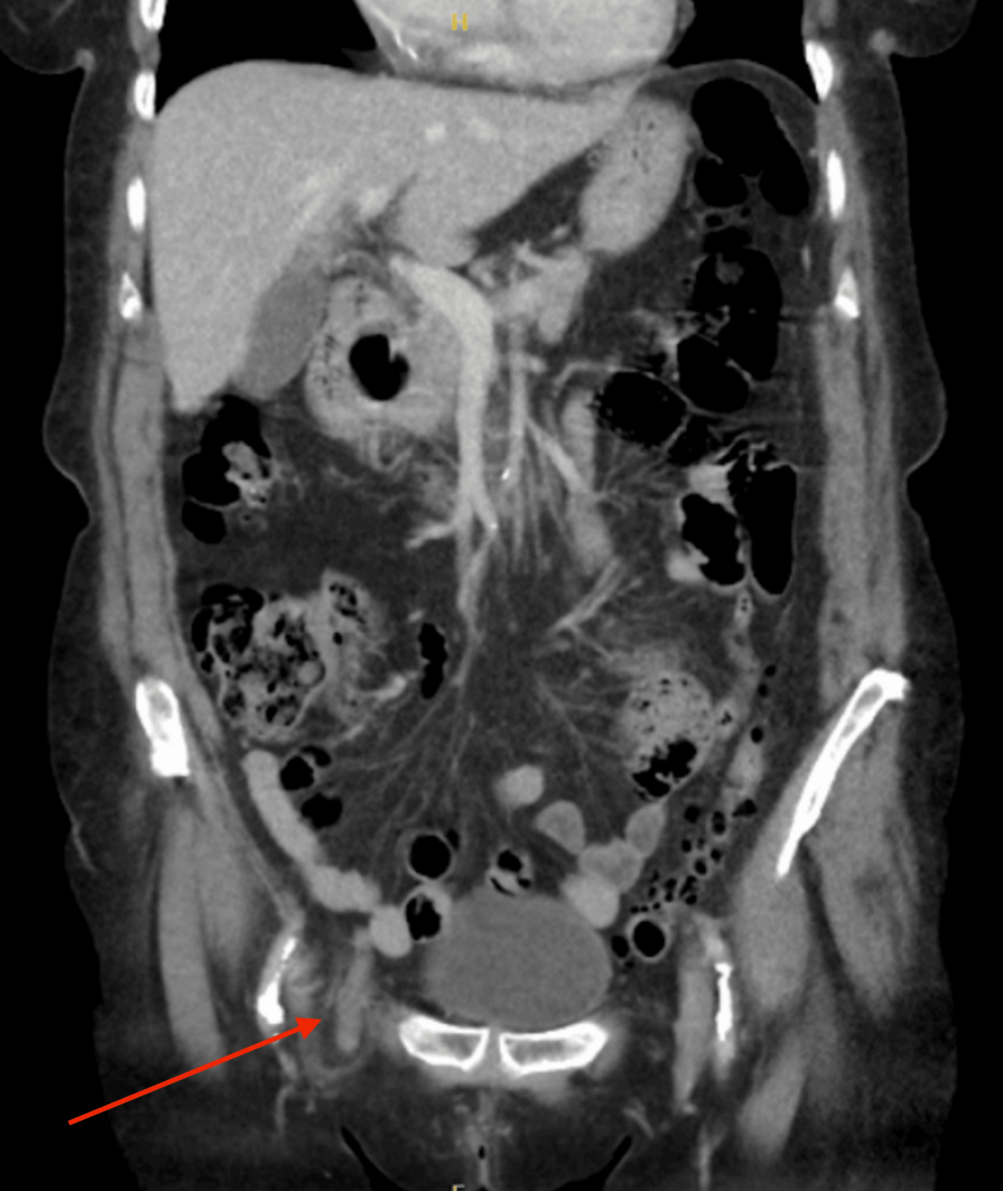
A coronal section of a CT scan showing an inflamed appendix within a right-sided femoral hernia, indicated by an arrow

A computed tomography (CT) scan confirmed the presence of a right femoral hernia containing the appendix (Figures 1-2). The appendix measured 100 mm in length and 11 mm in anteroposterior diameter, with the majority of its proximal portion remaining intra-abdominal. The distal aspect of the appendix within the hernial sac appeared inflamed, consistent with appendicitis.

Outcome and follow-up 

**Figure 3:**
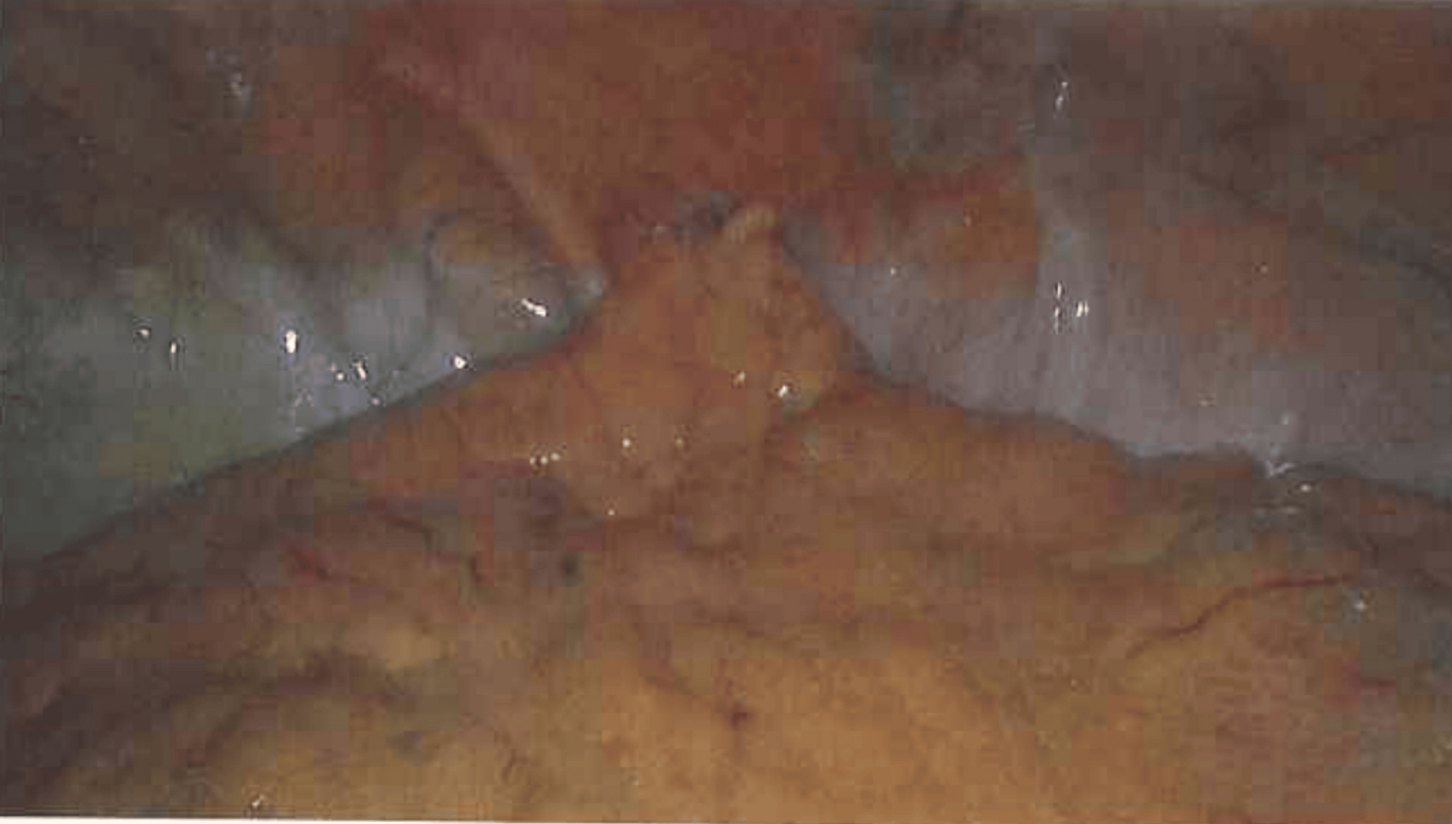
Intraoperative laparoscopic imaging showing an appendix with the omentum within the femoral hernia

**Figure 4:**
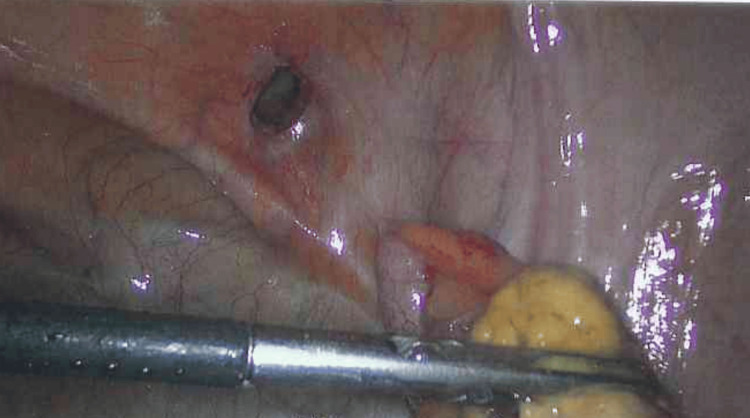
Intraoperative laparoscopic image showing the hernia defect, following reduction and removal of the appendix

As a result of these findings, the patient underwent diagnostic laparoscopy and groin exploration, with a plan for appendectomy as required. Intra-operatively, an inflamed appendix along with omentum incarcerated within the femoral hernial sac was identified (Figure 3). A laparoscopic appendectomy was performed (Figure 4), followed by open exploration of the groin via a low-approach. The femoral hernial sac was excised, and the femoral ring was repaired with interrupted nonabsorbable sutures. Histopathology later revealed focal areas of fat necrosis in the mesoappendix with no obvious evidence of acute transmural inflammation, suggesting secondary inflammation instead of primary infective pathology [6].

The patient had an excellent postoperative course and was discharged the following day, highlighting the importance of prompt diagnosis and early surgical intervention.

## Discussion

The patient presented with minimal symptoms despite having an inflamed appendix within a femoral hernia. This presentation is consistent with prior literature, where many cases of De Garengeot hernia with appendiceal inflammation lack the classical symptoms of infective appendicitis, such as right lower quadrant pain, fever, or systemic signs [7]. The confined anatomical position of the appendix within the hernial sac may localise the inflammatory process, preventing the development of generalised peritonitis [8].

Importantly, appendiceal inflammation in the context of a femoral hernia may be secondary to mechanical factors rather than primary appendicitis. Incarceration at the femoral ring can lead to compression of the appendix, resulting in compromised venous drainage, ischemia, and subsequent inflammatory changes [6,9]. In many reported cases, histopathology revealed only ischemic or inflammatory changes without true suppurative appendicitis, supporting the hypothesis of secondary inflammation due to strangulation [6].

The diagnosis of a De Garengeot hernia can be challenging. Distinguishing a femoral hernia from other groin pathologies relies primarily on clinical examination, yet this can be surprisingly unreliable. In one study of 278 patients, clinical diagnosis correctly identified only 39% of femoral hernias, compared to 85% of indirect inguinal and 64% of direct inguinal hernias [10].

On physical examination, the presence of a tender, irreducible groin mass below the inguinal ligament often suggests a femoral hernia. However, the unusual contents, such as the appendix, are difficult to ascertain clinically.

Ultrasound and cross-sectional imaging play a crucial role when the diagnosis is uncertain, particularly in women, obese patients, or atypical presentations. Dynamic ultrasound for femoral hernias demonstrates a sensitivity of 80% and specificity of 88%, with a negative predictive value of 92% [11]. However, operator dependence and variable acoustic windows can limit accuracy. Computed tomography (CT) offers higher anatomical resolution; it not only confirms the presence of a femoral sac but also differentiates its contents (e.g., omentum, bowel, and appendix) and detects secondary signs of strangulation or inflammation [12]. In this case, initial ultrasound was helpful, but CT imaging was essential in confirming the diagnosis and identifying signs of appendiceal inflammation [13].

Regarding management, elective femoral hernia repair in a clean field strongly favours prosthetic mesh reinforcement, either via an open plug-and-patch technique or a laparoscopic approach (TEP/TAPP) [14]. This preference for mesh repair originates from its consistently lower recurrence rates when compared to suture repair [15]. International guidelines (WSES) endorse mesh prostheses in incarcerated groin hernias without gross contamination, reporting no significant increase in infection or morbidity and a marked reduction in recurrence (grade 1A) [14].

Conversely, synthetic mesh carries a heightened risk of infection in contaminated or "clean-contaminated" fields, such as when appendiceal inflammation is present. Case series of appendicitis within femoral hernias advise against prosthetic materials and recommend primary tissue repair [16].

In the presented case of a De Garengeot hernia, combined laparoscopic appendectomy followed by open low‐approach herniorrhaphy with interrupted nonabsorbable sutures was appropriately chosen to minimise prosthesis‐related infectious risk.

## Conclusions

In summary, this case of a De Garengeot hernia illustrates the diagnostic challenges associated with groin hernias containing atypical contents and the clinical utility of CT scanning. Additionally, it reinforces the need to consider secondary appendiceal inflammation in cases of groin hernias, instead of primary infectious appendicitis. Finally, it highlights the rationale for primary tissue repair in "clean-contaminated" fields such as this.
